# PlantOrDB: a genome-wide ortholog database for land plants and green algae

**DOI:** 10.1186/s12870-015-0531-4

**Published:** 2015-06-26

**Authors:** Lei Li, Guoli Ji, Congting Ye, Changlong Shu, Jie Zhang, Chun Liang

**Affiliations:** Department of Automation, Xiamen University, Fujian, 361005 China; Department of Biology, Miami University, Oxford, OH 45056 USA; State Key Laboratory for Biology of Plant Diseases and Insect Pests, Institute of Plant Protection, Chinese Academy of Agricultural Sciences, Beijing, 100193 China; Innovation Center for Cell Signaling Network, Xiamen University, Xiamen, Fujian 361005 China

**Keywords:** Homolog, Ortholog, Paralog, Database, Land plants, Green algae, Gene family, PlantOrDB

## Abstract

**Background:**

Genes with different functions are originally generated from some ancestral genes by gene duplication, mutation and functional recombination. It is widely accepted that orthologs are homologous genes evolved from speciation events while paralogs are homologous genes resulted from gene duplication events.With the rapid increase of genomic data, identifying and distinguishing these genes among different species is becoming an important part of functional genomics research.

**Description:**

Using 35 plant and 6 green algal genomes from Phytozome v9, we clustered 1,291,670 peptide sequences into 49,355 homologous gene families in terms of sequence similarity. For each gene family, we have generated a peptide sequence alignment and phylogenetic tree, and identified the speciation/duplication events for every node within the tree. For each node, we also identified and highlighted diagnostic characters that facilitate appropriate addition of a new query sequence into the existing phylogenetic tree and sequence alignment of its best matched gene family. Based on a desired species or subgroup of all species, users can view the phylogenetic tree, sequence alignment and diagnostic characters for a given gene family selectively. PlantOrDB not only allows users to identify orthologs or paralogs from phylogenetic trees, but also provides all orthologs that are built using Reciprocal Best Hit (RBH) pairwise alignment method. Users can upload their own sequences to find the best matched gene families, and visualize their query sequences within the relevant phylogenetic trees and sequence alignments.

**Conclusion:**

PlantOrDB (http://bioinfolab.miamioh.edu/plantordb) is a genome-wide ortholog database for land plants and green algae. PlantOrDB offers highly interactive visualization, accurate query classification and powerful search functions useful for functional genomic research.

**Electronic supplementary material:**

The online version of this article (doi:10.1186/s12870-015-0531-4) contains supplementary material, which is available to authorized users.

## Background

Genes with different functions are originally generated from some ancestral genes by gene duplication, mutation and functional recombination. It is widely accepted that orthologs are homologous genes evolved from speciation events while paralogs are homologous genes resulted from gene duplication events [1-3]. With the rapid increase of genomic data, identifying and distinguishing these genes among different species is becoming an important part of functional genomics research. In the past, some people considered genes with same or similar functions in different species to be orthologs whereas others were trying to identify orthologs by similarity among gene sequences. However, ortholog genes in different species do not always keep the same functions, and the most similar gene sequences are not always orthologs [4]. It is getting more complicated as speciation and duplication events can occur alternately. As shown in Additional file 1: Figure S1 A, orthologs are reflexive because, as an example, *Arth-A1* is an ortholog of *Orsa-A1* and vise versa. Secondly, orthologs are non-transitive: *Arth-A1* is an ortholog of *Orsa-A1* and *Arth-A2* is an ortholog of *Orsa-A1*, but *Arth-A1* and *Arth-A2* are not orthologs. Thirdly, orthologs do not always have one-to-one relationship. Sometimes, they have one-to-many or many-to-many relationship due to alternation of duplication and speciation. For example, both *Arth-A1* and *Arth-A2* in Arabidopsis have a many-to-many ortholog relationship with *Orsa-A1* and *Orsa-A2* in rice, whereas *Orsa-B* in rice has a one-to-many ortholog relationship with *Arth-B1* and *Arth-B2* in Arabidopsis. Finally, there are two types of paralogs: in-paralogs and out-paralogs because duplication and speciation can occur alternately. In Additional file 1: Figure S1 A, there are three pairs of in-paralogs: *Arth-A1* and *Arth-A2*, *Orsa-A1* and *Orsa-A2*, and *Arth-B1* and *Arth-B2*, all of which are result of duplication. An out-paralog relation exists between any gene from *Arth-A1*, *Arth-A2*, *Orsa-A1* and *Orsa-A2* and any gene from *Arth-B1*, *Arth-B2*, and *Orsa-B*, which is result of either duplication-speciation or duplication-speciation-duplication events. With regard to sequence similarity, in general, a gene sequence is more similar to its in-paralogs than its orthologs, while it is more similar to its orthologs than its out-paralogs. As shown in Additional file 1: Figure S1 B, for example, *Arth-A1* has the shortest distance in sequence similarity to its in-paralog *Arth-A2,* the intermediate distance to its ortholog *Orsa-A1*, and the longest distance to its out-paralog *Arth-B1*.

Unfortunately, orthology is difficult to confirm by experimental methods. By far, there are two strategies to infer orthologs: phylogenetic methods (also known as tree-based methods) [5] and pairwise alignment methods (also known as graph-based methods) [6, 7]. Phylogenetic methods include 4 basic steps: (1) clustering of homologous genes into gene families, (2) multiple sequence alignment for each gene family, (3) generation of phylogenetic tree from multiple sequence alignment, and (4) identification of evolutionary events (i.e., duplication and/or speciation) to determine orthologs and paralogs. Limited by the accuracy of these individual steps, there are more or less errors in the final results using phylogenetic methods [8]. Particularly, phylogenetic methods often demand a lot of computational resources and such demands will increase exponentially when dealing with rapidly growing genome-wide protein sequences data. To address these challenges, pairwise alignment methods were developed to utilize less computational resources [6-8]. Essentially, it assumes that the most similar gene pairs between the genomes of two species are basic ortholog pairs. This theoretical foundation for pairwise alignment methods is not inarguable, due to the fact that most similar gene sequences in different species are not always orthologs [4]. Nevertheless, pairwise alignment methods are still being used because of low demands of computational resources [6, 7, 9]. There are two basic procedures for pairwise alignment methods: (1) infer basic ortholog pairs by a graph construction step: Reciprocal Best Hit (RBH) approach, also known as Bidirectional Best Hit (BBH) approach, and (2) merge related orthologs into gene clusters by a clustering step [7].

So far, a few ortholog databases have been generated, including OrthologID [10] (http://nypg.bio.nyu.edu/orthologid), InParanoid8 [7] (http://inparanoid.sbc.su.se/), Isobase [11] (http://groups.csail.mit.edu/cb/mna/isobase), CEG [12] (http://cefg.uestc.edu.cn/ceg/home.html),OrthoDB [13] (http://orthodb.org/), PhylomeDB4 [14] (http://phylomedb.org/), eggNOG 4.0 [15] (http://eggnog.embl.de/version_4.0.beta), EnsemblPlants (http://plants.ensembl.org/) [9] and PLAZA 3.0 [16] (http://bioinformatics.psb.ugent.be/plaza/versions/plaza_v3_dicots/). These databases are somewhat different in theoretical foundation, data sources, database functions, search capacity and information display methods. For instance, OrthologID and PhylomeDB4 adopted phylogenetic methods whereas PLAZA 3.0, OrthoDB, eggNOG 4.0, EnsemblPlants and InParanoid8 utilized pairwise alignment methods. OrthologID covers 5 plant species, PLAZA 3.0 includes 31 plant species and EnsemblPlants contains 38 plant species. CEG contains 16 bacterial species. OrthDB, eggNOG 4.0, PhylomeDB, Inparanoid8 include 1,367, 3,686, 1,059 and 100 species respectively, but all of them cover multiple kingdoms. Some databases, such as OrthologID, do not allow users to browse and retrieve all gene families and their search capabilities are very limited. Some databases do not permit users to upload their query sequences for analysis (e.g., Isobase and PhylomeDB) or provide a very preliminary BLAST report for the query sequences (e.g., CEG, PLAZA 3.0, EnsemblPlants and Inparanoid8). Some databases have rudimentary, less interactive web interfaces for result display. For instance, CEG and Isobase display result in text format while OrthoDB and eggNOG 4.0 displays sequence alignments in FASTA format. PLAZA 3.0, EnsemblPlants and PhylomeDB4 have built highly graphical and interactive interfaces. As more genomic data are rapidly accumulated, an emerging challenge for all databases is how to process and display large datasets accurately and effectively. For example, PLAZA 3.0 fails to display multiple sequence alignment for gene families with over 1,000 gene members and cannot show phylogenetic tree for gene families with more than 700 members.

Here, we present PlantOrDB, a genome-wide ortholog database that is developed using a phylogenetic method and contains over 1.5 million protein sequences from 35 land plants and 6 green algae. PlantOrDB provides data browsing capability that enables users to navigate and filter individual genes and gene families easily. It also offers robust search functions that allow name and ID search for individual gene and gene families, as well as keyword search for functional gene annotation (e.g., GO, KEGG, EC, Panther and PFAM). PlantOrDB provides users with highly interactive web interfaces for close examination of individual protein sequences, homolog gene families, phylogenetic trees, speciation/duplication events, multiple sequence alignment, and diagnostic characters that define each gene family’s character (amino acid) attributes. For any given gene, a user can infer its orthologs, in-paralogs and out-paralogs through our highly interactive web interfaces that provide integrated visualization of phylogenetic tree, multiple sequence alignment, speciation/duplication events and diagnostic characters. In particular, PlantOrDB lets users to explore all orthologs for a given gene, which are determined by RBH-based pairwise alignment method that conducts and utilizes all-against-all BLAST search for 35 plant and 6 algal species. By adopting RBH-based approach, PlantOrDB considers all RBH gene pairs in any of two different species as ortholog pairs. Using state-of-the-art web technologies, PlantOrDB is capable to show phylogenetic trees and sequence alignments for gene families with a large number of gene members interactively and smoothly. Moreover, PlantOrDB allows users to upload their own query sequence that can be anchored to the best matched gene family, with proper positions in the relevant phylogenetic tree and protein sequence alignment.

## Construction and content

### Data sources

The data source for PlantOrDB is Phytozome v9 (http://www.phytozome.net/). We extracted all 1,530, 047 protein sequences from 35 plant and 6 algal genomes. The evolutionary relationship among these 35 land plants and 6 green algae species is shown in Additional file 2: Figure S2.

### System architecture

The whole system is composed of a MySQL database, two Perl-based data processing pipelines and AJAX-based PHP web interfaces. One data processing pipeline is used to pre-build homolog gene families, identify orthologs and dump the resultant data into the database (we refer to this pipeline as “*pre-built*” pipeline thereafter), and the other one is for on-the-fly classification of the query sequence uploaded online by a user into its best matched gene family (we refer to this pipeline as “*on-the-fly*” pipeline thereafter). The “*pre-built*” pipeline clusters protein sequences into homolog gene families. Then, it creates multiple alignments, builds phylogenetic trees, identifies speciation/duplication events, and detects diagnostic characters for all gene families. After a user submits a query sequence online, the “*on-the-fly*” pipeline will find the best matched gene family for the query, and insert it into the proper places within the existing phylogenetic tree and peptide alignment of the best matched gene family.

As shown in Additional file 3: Figure S3, our “*pre-built*” pipeline integrates both phylogenetic method and pairwise alignment method, which are composed of 5 and 2 steps, respectively. In the phylogenetic method, the 5 steps are: *Homolog Gene Family Builder*, *Multiple Sequence Alignment (MSA) Generator*, *Phylogenetic Tree Creator, Speciation/Duplication Events Identifier* and *Diagnostic Character Identifier*. Clearly, this part of pipeline follows the basic strategy and procedures used for phylogenetic method or tree-based ortholog identification [4, 10], with our own implementations, modifications and improvements.

Firstly, *Homolog Gene Family Builder* clusters all amino acid sequences into gene families based on sequence similarity search results using BLAST [17]. Here, there are three steps involved: *all-against-all BLAST search*, *BLAST result filtration* and *gene family creation*. In order to get the *all-against-all BLAST search* results, we developed a Perl-based program suitable for multiple cores in a standard single server and shortened BLAST execution time tremendously. For *BLAST result filtration*, we adopted an e-value threshold and overlap region rule when filtering the *all-against-all BLAST search* results. For any two gene sequences, either from the same species or different species, if their BLAST e-value is within the 1e-10 cutoff (e-value threshold) and the overlapped region is more than 80 % of the longer sequence (overlap region rule), they will be treated as homologous genes. If a gene is homologous to a gene within a gene family, this gene will be considered as a member of that family. *Homolog Gene Family Builder* picks randomly a gene (sequence), finds all relative genes recursively from the *all-against-all BLAST search* outputs and generates one gene family. Then, *Homolog Gene Family Builder* picks randomly another gene, which is not listed as a gene within any established gene family so that one gene only belongs to one gene family, and iterates the whole process to assign every gene to an appropriate gene family. The minimum gene number for a given gene family is 2, which means that singleton sequences will be discarded at the current version of our database.

Next, *MSA Generator* conducts multiple sequence alignment for individual gene families using MAFFT 7.0 [18]. Many software tools such as MAFFT [18] and ClustalW [19] are able to complete multiple sequence alignment tasks with reliable accuracy. In particular, MAFFT 7.0 has an unique option “*--add*” [20] that can add a new unaligned sequence into an existing multiple sequence alignment. This unique feature is essential for our “*on-the-fly*” pipeline, which needs to classify a query sequence uploaded online by a user temporarily without re-constructing multiple sequence alignments. That is why MAFFT 7.0 is advantageous here over other multiple sequence alignment tools.

As shown in Additional file 3: Figure S3, the third step in the phylogenetic method of our “*pre-built*” pipeline is *Phylogenetic Tree Creator* that uses multiple sequence alignments to build phylogenetic trees. Here, we adopted FastTree2 [21] as our Tree Builder. Other tools like PAUP* [22], PHYLIP [23], RAxML [24] and PhyML [25] are also popular for creating phylogenetic trees. Since some of our gene families contain over 10,000 genes, we have put tremendous efforts in experimenting different tree building tools that can be scaled up to process huge gene families. Based on approximately maximum-likelihood method, FastTree2 was designed to process huge multiple sequence alignments efficiently, using reasonable amount of memory without sacrificing the quality of phylogenetic trees. FastTree2 proves to be from 100 to 1,000 times faster than PhyML 3.0 or RAxML 7 for large sequence alignments [21]. That was why we selected FastTree2 for PlantOrDB.

The fourth step in the phylogenetic method of our “*pre-built*” pipeline is *Speciation/Duplication Event Identifier* which can identify the evolutionary events, either speciation or duplication, for every node in phylogenetic trees. We have implemented the Speciation versus Duplication Inference (SDI) algorithm [26] in Perl. This Perl program utilizes the species phylogenetic tree (see Additional file 2: Figure S2) as the reference tree.

Finally, the fifth step in the phylogenetic method of our “*pre-built*” pipeline is *Diagnostics Character Identifier*, which extracts all diagnostic characters for all gene families. Similar to OrthologID [10], our pipeline determines diagnostic characters that characterize or define each gene set (or group) using both multiple sequence alignments and phylogenetic trees. As shown in Additional file 4: Figure S4, there are two types of diagnostic characters to differentiate groups in PlantOrDB: pure and private. For a node in Additional file 4: Figure S4, we define all of its child sequences as its clades. Both pure and private diagnostic characters are exclusively appeared in its clades. The difference is that the pure diagnostic characters are shared by all members within a clade whereas the private diagnostic characters are shared by some members within a clade. We have implemented CAOS algorithm [27] in Perl for *Diagnostics Character Identifier*.

The pairwise alignment method part of “*pre-built*” pipeline consists of 2 steps: *All-against-all Blast* and *Ortholog Identifier*. Similar to previous studies [28-30], we extracted RBH (reciprocal best hit) records from *all-against-all BLAST search* results. The classical pairwise alignment methods contain more steps in addition to ortholog pair identification, including deletion of false-positive ortholog pairs, addition of in-paralogs into ortholog pairs, and merging of closely related ortholog pairs to form homolog gene families [6, 7]. Because we had already built homolog gene families, multiple sequence alignments and phylogenetic trees and identified speciation/duplication events by phylogenetic method, it is unnecessary for us to rebuild gene families by pairwise alignment method. Therefore, we just identified orthologs for all genes using RBH-based pairwise alignment method in PlantOrDB.

In comparison with the “*pre-built*” pipeline, our “*on-the-fly*” pipeline is much simpler because its major function is to classify query sequences uploaded online from users into the existing gene families. Traditionally, the only way to plug a new query sequence into an existing gene family is to add this sequence into its best matched family, redo multiple sequence alignment, and reconstruct the phylogenetic tree using the new alignment result. Fortunately, CAOS algorithm can be used to not only extract the character attributes of a given gene family and compute its diagnostic character states, but also add a new sequence into an existing phylogenetic tree properly after working with MAFFT 7.0 that can add a query sequence into the existing alignment without reconstructing the whole multiple sequence alignment [31]. When a user submits a query sequence online, the backend “*on-the-fly*” pipeline will be invoked to process the sequence by the following steps: (1) determine the best matched gene family by BLAST [17], (2) align the query sequence into the existing multiple sequence alignment of the best matched gene family using MAFFT 7.0 (i.e., −−add option), and (3) insert the aligned sequence into the phylogenetic tree of the best matched family by CAOS program. As shown in Additional file 5: Figure S5, in order to insert the query sequence into the existing phylogenetic tree of its best matched gene family in an appropriate position, CAOS program uses this existing tree as a guide tree, searches the matches between query sequence and diagnostic characters of nodes from the root to branches of the guide tree, and determines the proper node position for the query sequence. It is worthy of mentioning that OrthologID had a similar pipeline for online query classification where BLAST, rather than MAFFT 7.0, was utilized in the aforementioned step (2).

#### Database, file system and web interface implementation

We created a MySQL database to store all indexed information, including summary information of homolog gene families, association relationship between protein sequences and gene families, gene orthologs and functional annotations of individual genes obtained from Phytozome v9. PlantOrDB also generated a lot of files to store detailed information of individual gene families. They are family alignment files, family tree files, diagnostic characters files and character attribute files. Web interfaces were implemented in PHP (http://php.net/), with JavaScript and HTML (http://www.w3.org/). PlantOrDB utilized AJAX (Asynchronous JavaScript and XML) technology to dynamically load and refresh the websites, greatly reducing the loading time and enhancing web interface usability. Based on the jQuery (http://jquery.com/) JavaScript framework, PlantOrDB constructed a set of highly interactive web interfaces. PlantOrDB also used other open source JavaScript plug-ins, e.g., Highcharts (http://www.highcharts.com/) and JTable (http://www.jtable.org/), to display various data retrieved from the aforementioned files and the database. Our web interfaces are compatible with different internet browsers like Mozilla Firefox (8.0 or above), Google Chrome, Safari and Internet Explorer (9.0 or above), and have been tested with different Operation Systems including Macintosh, Linux and Windows

### Utility

Overall, we have extracted 1,530,047 peptide sequences for 41 genomes (i.e., 35 land plant and 6 green algae species) from Phytozome v9 (http://www.phytozome.net/). Among them, 1,291,670 amino acid sequences have been clustered into 49,355 homolog gene families. In particular, 22 homolog gene families have more than 1,000 family members. Moreover, PlantOrDB has taken advantages of Phytozome v9 gene annotation files that contain KEGG EC, KEGG Ortholog, KOG, Panther and PFAM information and parsed them into our backend MySQL database, which can be queried through our web interfaces.

**Fig. 1:**
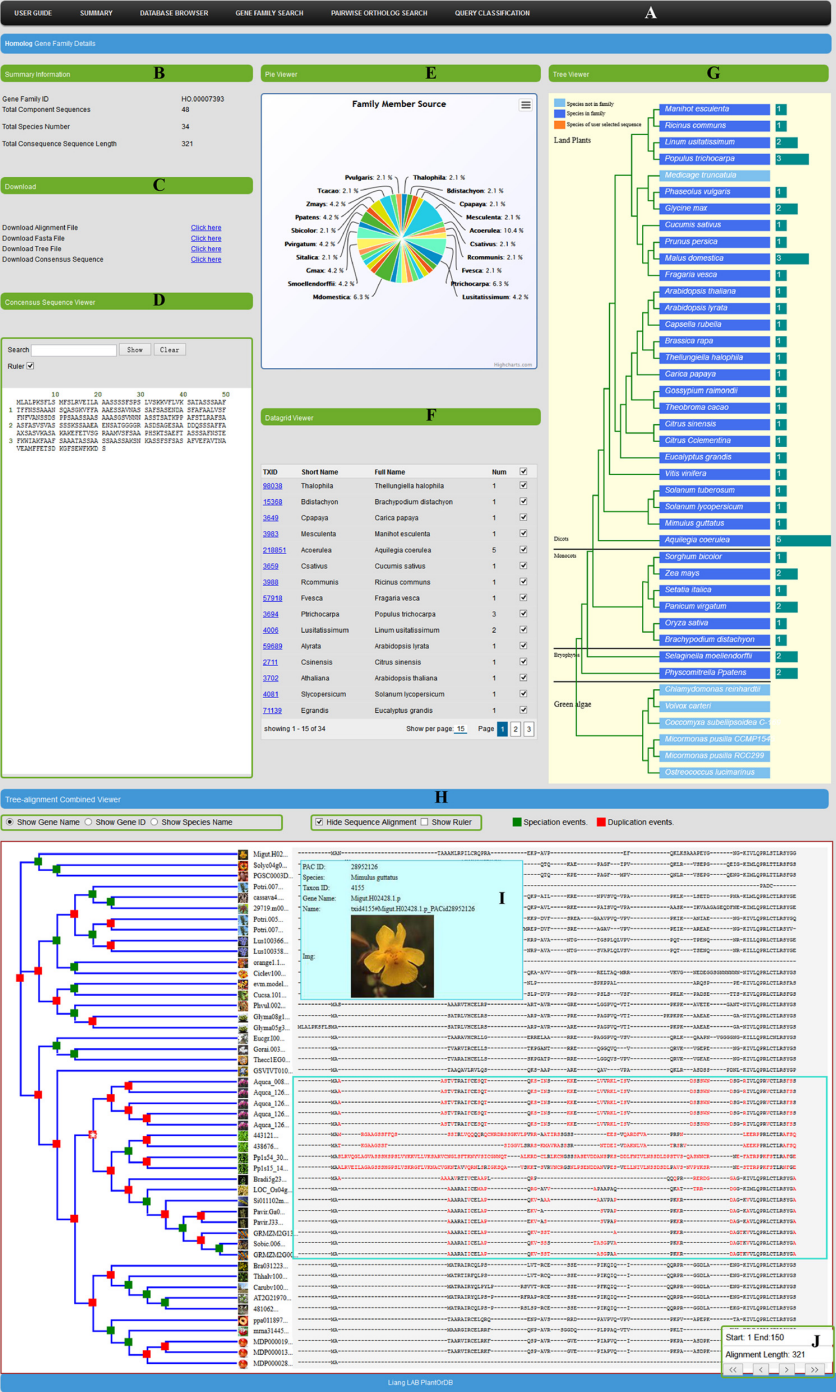
The snapshots of the main web interface of PlantOrDB. Panel **a**: the navigation bar of PlantOrDB. Panel **b**: Summary Information. Panel **c**: Download. Panel **d**: Consensus Sequence Viewer. Panel **e**: Pie Viewer. Panel **f**: Datagrid Viewer. Panel **g**: Tree Viewer. Panel **h**: Tree-alignment Combined Viewer. Panel **i**: gene information panel. Panel **j**: navigation panel

The major web portal of PlantOrDB is divided into six components: *“USER GUIDE”*, *“SUMMARY”*, *“DATABASE BROWSER”*, *“GENE FAMILY SEARCH”*, *“PAIRWISE ORTHOLOG SEARCH”* and *“QUERY CLASSIFICATION”*, shown as in the navigation bar in Fig. 1a. The *“USER GUIDE”* is a tutorial page that helps users utilize and be familiar with our web interfaces. The *“SUMMARY”* has two submenus items: *“About PlantOrDB”* and *“Data Source”*, which provide a database overview and some descriptions of our data source respectively. The *“DATABASE BROWSER”* contains four submenus items: *“Gene Families”*, *“Protein Sequences”*, *“Gene Annotation”* and *“Individual Gene Sequence-Annotation Viewer”*. Through these items, users can navigate, browse, view and search both the summary and detailed information of gene families, protein sequences and their functional annotations. The *“GENE FAMILY SEARCH”* allows users to search homolog gene families by a gene family ID, full gene name and gene sequence ID. The *“PAIRWISE ORTHOLOG SEARCH”* allows users to search all RBH-pairwise-alignment-based orthologs for a given gene. After a user specifies a gene sequence ID, the interface will show an ortholog tree that contains all orthologs for the selected gene and their relevant orthologs recursively. Furthermore, the interface also can show the ortholog path between any two genes in the ortholog tree, which can describe how these two genes are linked through their orthologs. The *“QUERY CLASSIFICATION”* tab allows users to submit a single query sequence and classify it into an existing, best matched homolog gene family. The query sequence will be inserted into appropriate positions within the phylogenetic tree and multiple sequence alignment of the best matched gene family for interactive visualization.

As shown in Fig. 1, PlantOrDB provides a highly interactive web interface for each gene family that allows selective visualization of the phylogenetic tree, multiple sequence alignment, evolutionary events and diagnostic characters. Our major web interface has two panels: *“Homolog Gene Family Details”* and *“Tree-alignment Combined Viewer”*.

The *“Homolog Gene Family Details”* panel consists of *“Summary Information”* section (Fig. 1b), *“Download”* section (Fig. 1c), *“Consensus Sequence Viewer”* (Fig. 1d), *“Pie Viewer”* (Fig. 1e), *“Datagrid Viewer”* (Fig. 1f) and *“Tree Viewer”* (Fig. 1g). *“Summary Information”* section (Fig. 1b) shows gene family ID, total component sequences, total species number and consensus sequence length. *“Download”* section (Fig. 1c) enables users to download family alignment, sequences in FASTA format, phylogenetic tree and the consensus sequence. *“Consensus Sequence Viewer”* (Fig. 1d) shows the consensus sequence with a ruler and pattern search capability. *“Pie Viewer”* (Fig. 1e) shows all component species in a given gene family and their composition percentages (i.e., how many different gene sequences from the same species within a given gene family). From this pie chart, users can easily know species distribution of the current gene family: whether this is a family specific to a species, sub-group of all species, or all 41 species. *“Datagrid Viewer”* (Fig. 1f) provides more detailed information about species composition for a given gene family. It shows species taxon ID, abbreviated species name, full species name and the number of different genes from one species within a given gene family. The last column of *“Datagrid Viewer”* is a checkbox HTML element. By default, all the checkboxes are checked. When a user unchecks the checkbox for a certain species or sub-group of all species, the sequence alignment and phylogenetic tree parts for the unchecked species will be invisible. This unique feature allows users to focus on a desired species or subgroup of all species for selective view of the phylogenetic tree and sequence alignment within a given gene family. *“Tree Viewer”* (Fig. 1g) shows species composition information of gene family members for a given gene family, using species-based phylogenetic tree where the numbers of component gene family numbers are highlighted for each species.

There are two parts in our *“Tree-alignment Combined Viewer”* (Fig. 1h): phylogenetic tree on the left and multiple sequence alignment on the right. Within a phylogenetic tree, by default, gene names and species icons are used to label each leaf. When users move mouse over a gene name or species icon, a pop-up window that shows detailed information for the gene will be displayed (Fig. 1i). Moreover, users can change the default labelling mode by changing the radio buttons “*Show Gene Name*”, “*Show Gene ID*” and “*Show Species Name*”. Both the sequence alignment and a ruler that facilitates positioning can be turned on or off by two check boxes: “*Show/Hide Sequence Alignment*” and “*Show/Hide Ruler*”. There is a navigation bar floating on the bottom right side (Fig. 1j), which shows the total alignment length for a gene family and the positions of current aligned region, with four buttons for users to move the alignment to the left or right, at a normal or faster pace. Because we adopted AJAX to implement this web interface, we are able to show phylogenetic tree and sequence alignment smoothly for gene families with a large number of gene members. Our AJAX-based web interfaces just request and load a small part of data at one time, instead of pre-loading the whole data set for a gene family, greatly reducing the loading time when viewing huge gene families. Within a phylogenetic tree, each node is marked with a green or red rectangle. The green rectangle stands for a speciation event while the red rectangle for a duplication event, facilitating ortholog or paralog identification. Moreover, the part of the phylogenetic tree is also interactive: when users click on a node in the phylogenetic tree, alignment section will appear a light blue rectangle to surround and highlight all child sequences inside this clicked node. Then all diagnostic characters within the light blue rectangle will be highlighted in red. Clearly, visualizing diagnostic characters will be essential for validating the quality of multiple sequence alignments and phylogenetic trees.

**Fig. 2:**
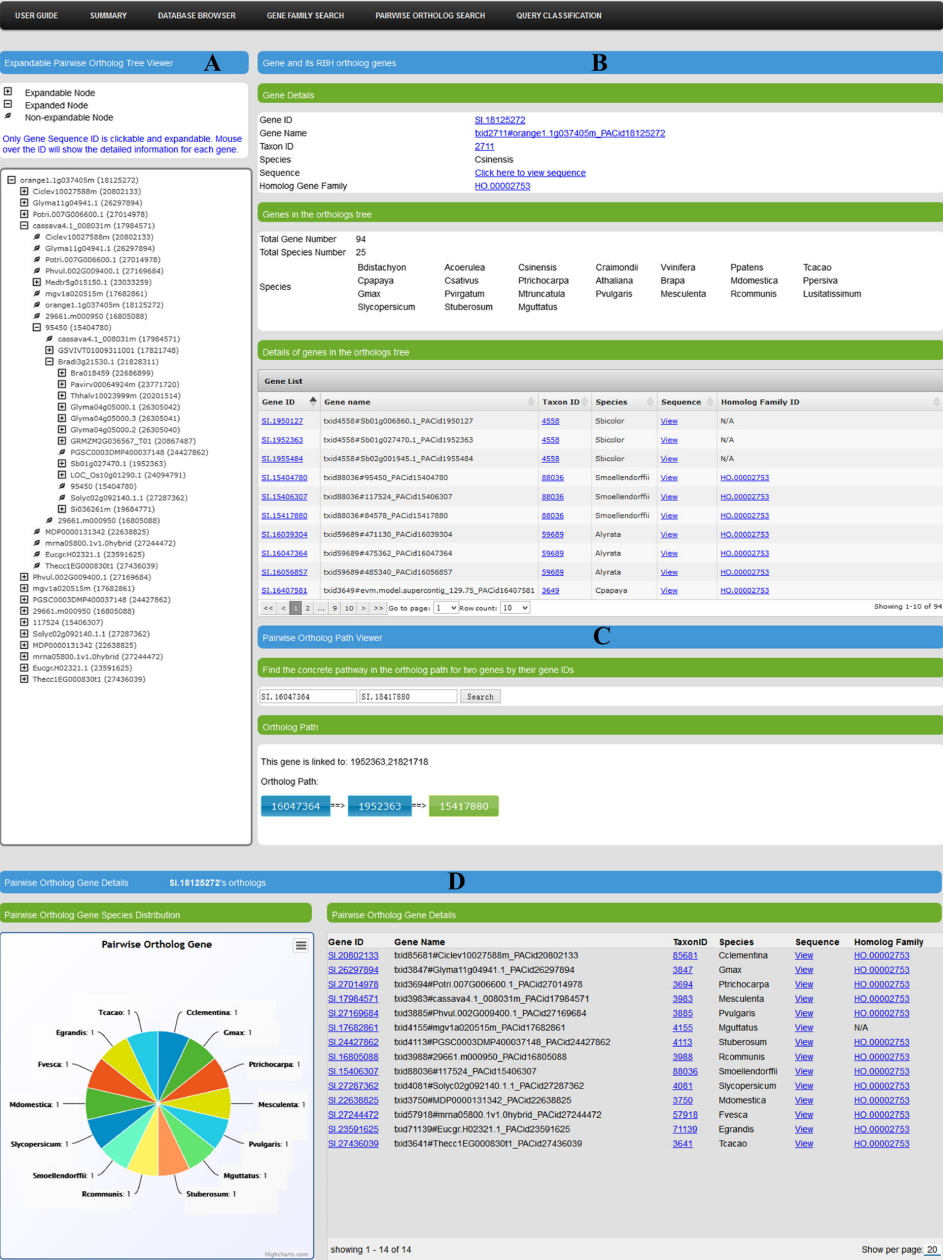
The snapshots of the ortholog gene web interface of PlantOrDB. Panel **a**: Expandable Pairwise Ortholog Tree Viewer. Panel **b**: Gene and Its RBH Ortholog Genes. Panel **c**: Pairwise Ortholog Path Viewer. Panel **d**: Pairwise Ortholog Gene Details

For a given gene, PlantOrDB provides not only its gene family, protein sequence alignment, phylogenetic tree and evolutionary (species/duplication) event information, but also gene sequence-annotation information, as shown in our *Individual Gene Sequence-Annotation Viewer* (see Additional file 6: Figure S6), and RBH-pairwise-alignment-based ortholog genes (see Fig. 2). As shown in Fig. 2, there are four parts for the ortholog interface. The first one is *“Expandable Pairwise Ortholog Tree Viewer”* (Fig. 2a), which shows all RBH-pairwise-alignment-based orthologs for a given gene and their relevant orthologs recursively, with the root node being the gene specified by a user. The second one is *“Gene and Its RBH Ortholog Genes”* (Fig. 2b), which provides details about the specific gene, its RBH-based ortholog genes, and other relevant genes within the ortholog tree. The third part is *“Pairwise Ortholog Path Viewer”* (Fig. 2c), which shows the concrete ortholog pathway between any two ortholog gene pair within the ortholog tree so that we know how these two genes are linked through their pairwise-alignment-based orthologs. This is a novel function that is not available in all aforementioned other databases. The fourth part is *“Pairwise Ortholog Gene Details”* (Fig. 2d), which presents a pie chart and data grid table to describe the species composition and detailed information of all pairwise-alignemnt-based orthologs for a given gene.

## Discussions

Although a few ortholog databases have been mentioned previously, we will focus on comparing the six plant centric ortholog databases: OrthologID, PLAZA 3.0, Inparanoid8, PhylomeDB4, EnsemblPlants and PlantOrDB in terms of their data amount, database functions and performance of user interfaces.

All of these six databases have conducted genome-scale ortholog identification for land plants. OrthologID contains 137,641 protein sequences for three plant species: *Arabidopsis thaliana*, *Oryza sativa* and *Populs trichocarpa*. PLAZA 3.0 collected 1,087,713 genes from 31 plants. EnsemblPlants utilized 690,172 genes from 21 plant species. Based on Phytozome v9, PlantOrDB has 1,530,047 genes from 35 plant and 6 green algal species. Although Inparanoid8 and PhylomeDB4 have much more species: 100 and 1,059 species respectively, they include species from different kingdoms. In terms of database functions, PlantOrDB and PhylomoDB4 developed a navigable browser that allows users to view and navigate the summary information of all gene families and individual protein sequences, which is not available in other aforementioned databases. In terms of search capability, PhylomeDB and OrthologID have very limited interfaces that only allow users to search by gene name. InParanoid8 allows users to search by species, gene family size or gene/protein ID. EnsemblPlants allows users to search by gene ID, species, synonyms and descriptions. Apparently, PlantOrDB and PLAZA 3.0 have better search functions because both of them permit users to search through their lists of individual genes and gene families by different ways. Moreover, PlantOrDB allows users to search gene families by gene functional annotation (e.g., GO, KEGG, EC, Panther and PFAM), which is not available in OrthologID, while PLAZA 3.0 only allows users to search GO terms.

As tree-based ortholog databases, OrthologID, PlantOrDB and PhylomeDB4 provide homolog gene families, multiple sequence alignments and phylogenetic trees. For both PlantOrDB and PhylomeDB4, users can infer ortholog relations from evolutionary events annotated in the phylogenetic trees in comparison with the species phylogenetic tree. Because OrthologID did not identify speciation/duplication events, it is actually difficult for user to identify the true orthologs in OrthologID. Different from the tree-based ortholog databases, InParanoid8, PLAZA 3.0 and EnsemblPlants are pairwise-alignment-based or graph-based ortholog databases. InParanoid8 generates homologous gene families that contain RBH-pairwise-alignment-based orthologs and their in-paralogs while EnsemblPlants provides RBH-pairwise-alignment-based orthologs and relevant in-paralogs for 20 monocot genomes. PlantOrDB is capable to show pairwise alignment orthologs by *Pairwise Ortholog Search* function for 35 plant and 6 algal species, which is not available in OrthologID and PhylomeDB4. Although PLAZA 3.0 is capable to show all orthologs for a given gene, PlantOrDB does a better job by providing more detailed information and useful visualization through *“Expandable Pairwise Ortholog Tree Viewer”*, *“Gene and Its RBH Ortholog Genes”*, *“Pairwise Ortholog Gene Details”* and a novel *“Pairwise Ortholog Path Viewer”* that shows how two genes are linked through their orthologs (see Fig. 2).

PLAZA 3.0 allows users to submit their own sequences to do BLAST against the whole database, and returns the blast report in text format. For a given query sequence, PhylomeDB4 will return all matched gene families by BLAST that meet the required E-value threshold. EnsemblPlants allows users to blast query sequence to up to 25 species, and then returns blast report in text format. InParanoid8 can show all matched genes and their homolog gene families for a given query. For a query sequence uploaded by users, both OrthologID and PlantOrDB will find the best matched gene family by BLAST, and then they insert the query sequence into appropriate positions of both the phylogenetic tree and multiple sequence alignment of the best matched gene family, without rebuilding the phylogenetic tree and multiple sequence alignment. OrthologID fails to identify node speciation/duplication events for query classification result. In comparison with other databases, PlantOrDB provides more informative analysis results and data visualization for the users’ query sequences.

It is clear that interactive graphical interfaces can provide more useful information than text results for biologists. When showing gene families and query sequence classification results, PlantOrDB provides integrated graphical web interfaces to show the phylogenetic tree and sequence alignment synergically and interactively. Furthermore, PlantOrDB’s AJAX-based interfaces are more dynamic and interactive than OrthologID's interfaces, by reducing greatly the loading time of the data and providing smooth transitions between navigations. PLAZA 3.0 offers a Java-based browser to view multiple sequence alignment, but it does not allow selective view of the partial alignments that focus on a desired subgroup of all species. PLAZA 3.0 also provides an independent Java-based phylogenetic tree viewer that has no connection with its multiple sequence alignment browser. To view both the phylogenetic tree and multiple sequence alignment from PLAZA 3.0, the java codes need to be downloaded into a client computer, which sometimes is prohibited by installed anti-virus software or rejected by online security systems. Moreover, both alignment and tree viewers in PLAZA 3.0 are not available for gene families with a large number of gene members. EnsemblPlants provides highly interactive web interfaces to show phylogenetic tree and alignment summary graphically, but fails to show alignment in details. PhylomeDB4 and PLAZA 3.0 show sequence alignments and phylogenetic trees on twoindependent web interfaces. In contrast, PlantOrDB has a seamlessly integrated interface, *Tree-Alignment Combined Viewer*, for viewing both a phylogenetic tree and relevant multiple sequence alignment simultaneously. The AJAX-based web interfaces in PlantOrDB perform well when displaying the phylogenetic tree and multiple sequence alignment for huge gene families, especially for those with over a thousand gene members. The AJAX technology can load a small part of data at one time, instead of pre-loading the whole data like OrthologID does. The AJAX-based web interfaces not only highly reduced the loading time but also made viewing larger gene families smooth. In particular, PlantOrDB offers selective visualization of phylogenetic tree and sequence alignment that can focus on a desired species or subgroup of all species, which is not available in other databases like PLAZA 3.0 and OrthologID. Furthermore, PLAZA 3.0, PhylomeDB4, EnsemblPlants and InParanoid8 do not show diagnostic characters, which are integrated with multiple sequence alignment and phylogenetic trees in the *Tree-alignment Combined Viewer* in PlantOrDB.

## Conclusion

Built on 35 plant and 6 green algal genomes released from Phytozome v9, PlantOrDB is a genome-wide ortholog database for land plants and green algae. The highly interactive web interfaces provided by PlantOrDB can display useful information on individual gene, and its homolog gene families and ortholog genes interactively and dynamically. Furthermore, PlantOrDB provides accurate query classification and useful data visualization of query sequences within phylogenetic tree and multiple sequence alignment, with powerful search functions useful for functional genomics research. On the other hand, some other databases such as PLAZA 3.0 and EnsemblPlants are able to provide many comparative genomics tools (e.g., collinear region plot and localization plot) that PlantOrDB currently does not offer. In the future, we will incorporate these tools into our database and make PlantOrDB more useful to the research community.

## Availability and requirements

The open-access database is available on (http://bioinfolab.miamioh.edu/plantordb). All data sets can be downloaded freely. We have tested our web interfaces using Google Chrome, Mozilla Firefox (8.0 or above) and Microsoft Internet Explorer (9.0 or above) under different Operation Systems including Macintosh, Linux and Windows. For the best visualization effect and performance, we recommend Mozilla FireFox and Google Chrome.

## Additional files

Additional file 1: Figure S1. Definitions of ortholog, in-paralog and out-paralog due to specification and duplication. An ancestral gene after duplication results in two in-paralogs: Gene A and Gene B. After speciation, Gene A generates two ortholog genes in Arabidopsis and rice, each of which after duplication results in two in-paralogs: Arth-A1 versus Arth-A2 and Orsa-A1 versus Orsa-A2, respectively. Any A gene (i.e., Arth-A1 and Arth-A2) in Arabidopsis has a many-to-many ortholog relationship with any A gene in rice (i.e., Orsa-A1 and Orsa-A2). After speciation, Gene B generates Orsa-B and its ortholog gene in Arabidopsis, which after duplication results in two in-paralogs: Arth-B1 and Arth-B2. Orsa-B has a one-to-many ortholog relationship with any B gene in Arabidopsis (i.e., Arth-B1 and Arth-B2). An out-paralog relation can be found between any A gene (i.e., Arth-A1, Arth-A2, Orsa-A1 and Orsa-A2) and any B gene (i.e., Arth-B1, Arth-B2, and Orsa-B). Additional file 2: Figure S2. The 35 land plant and 6 green algae species utilized in PlantOrDB. Additional file 3: Figure S3. The structure and work flow of the bioinformatics pipeline to pre-build homolog gene families and identify orthologs. Additional file 4: Figure S4. Pure and private diagnostic characters detected and utilized by CAOS algorithm. Additional file 5: Figure S5. The graphic representation of the core CAOS algorithm. Additional file 6: Figure S6. The web interface of Individual Gene Sequence-Annotation Viewer.

## Abbreviations

RBH: Reciprocal best hit
AJAX: Asynchronous JavaScript and XML
BBH: Bidirectional best hit
KEGG: Kyoto encyclopedia of genes and genomes
GO: Gene ontology
EC: Enzyme commission
