# Supplementary figures and images for: PlantOrDB: a genome-wide ortholog database for land plants and green algae
Source: BMC Plant Biol. 2015 Jun 26;15:161. doi: 10.1186/s12870-015-0531-4 (PMC4481079; doi:10.1186/s12870-015-0531-4)

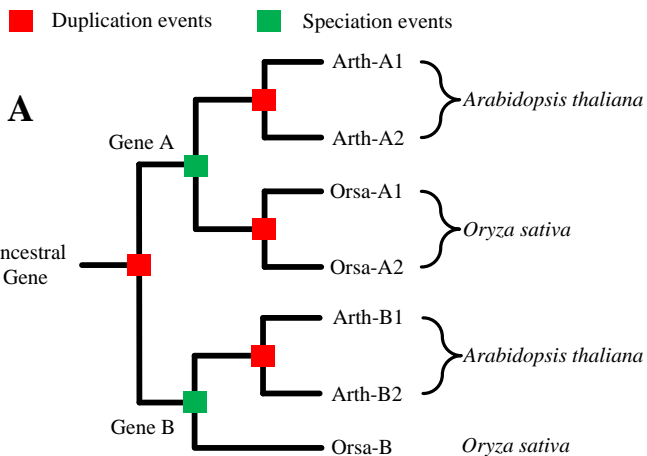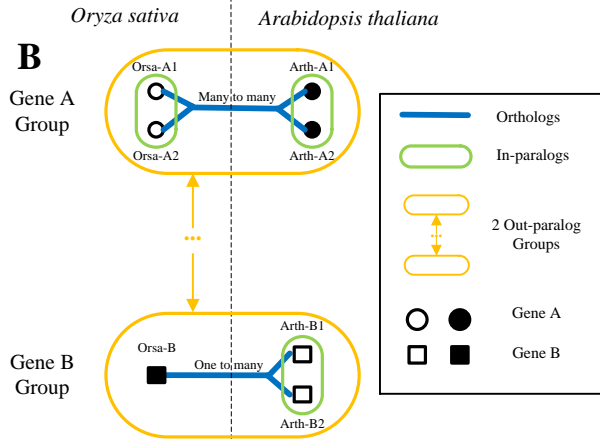

Supplement: Additional file 1: Figure S1. — Definitions of ortholog, in-paralog and out-paralog due to specification and duplication. An ancestral gene after duplication results in two in-paralogs: Gene A and Gene B. After speciation, Gene A generates two ortholog genes in Arabidopsis and rice, each of which after duplication results in two in-paralogs: Arth-A1 versus Arth-A2 and Orsa-A1 versus Orsa-A2, respectively. Any A gene (i.e., Arth-A1 and Arth-A2) in Arabidopsis has a many-to-many ortholog relationship with any A gene in rice (i.e., Orsa-A1 and Orsa-A2). After speciation, Gene B generates Orsa-B and its ortholog gene in Arabidopsis, which after duplication results in two in-paralogs: Arth-B1 and Arth-B2. Orsa-B has a one-to-many ortholog relationship with any B gene in Arabidopsis (i.e., Arth-B1 and Arth-B2). An out-paralog relation can be found between any A gene (i.e., Arth-A1, Arth-A2, Orsa-A1 and Orsa-A2) and any B gene (i.e., Arth-B1, Arth-B2, and Orsa-B). [file 12870_2015_531_MOESM1_ESM.pdf]

Land Plants

Dicots

Monocots

Bryophytes

Green algae

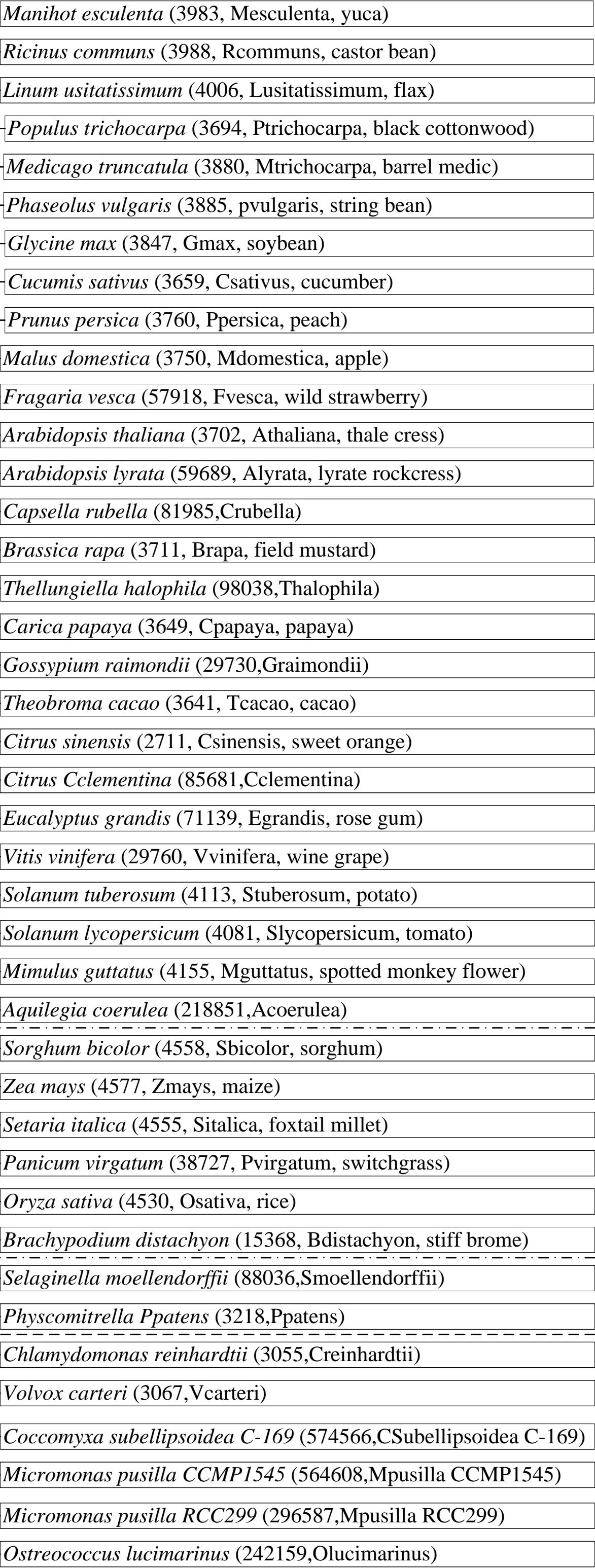

Supplement: Additional file 2: Figure S2. — The 35 land plant and 6 green algae species utilized in PlantOrDB. [file 12870_2015_531_MOESM2_ESM.pdf]

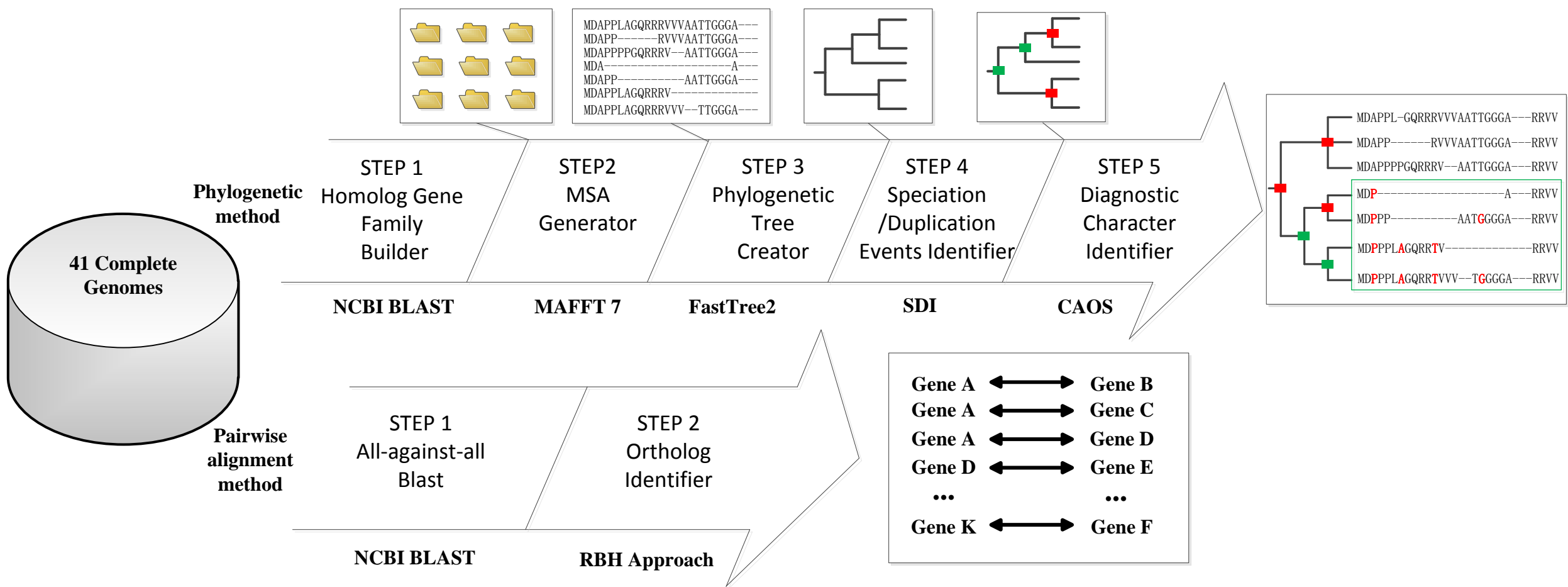

Supplement: Additional file 3: Figure S3. — The structure and work flow of the bioinformatics pipeline to pre-build homolog gene families and identify orthologs. [file 12870_2015_531_MOESM3_ESM.pdf]

Position      1   2   3   4   5   6   7   8   9

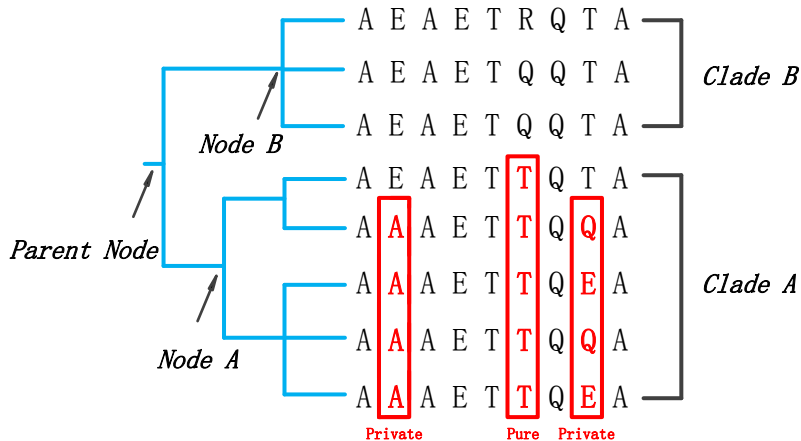

Supplement: Additional file 4: Figure S4. — Pure and private diagnostic characters detected and utilized by CAOS algorithm. [file 12870_2015_531_MOESM4_ESM.pdf]
