# Supplementary material for: PlantOrDB: a genome-wide ortholog database for land plants and green algae
Source: BMC Plant Biol. 2015 Jun 26;15:161. doi: 10.1186/s12870-015-0531-4 (PMC4481079; doi:10.1186/s12870-015-0531-4)
Supplement: Additional file 5: Figure S5. — The graphic representation of the core CAOS algorithm. [file 12870_2015_531_MOESM5_ESM.pdf]

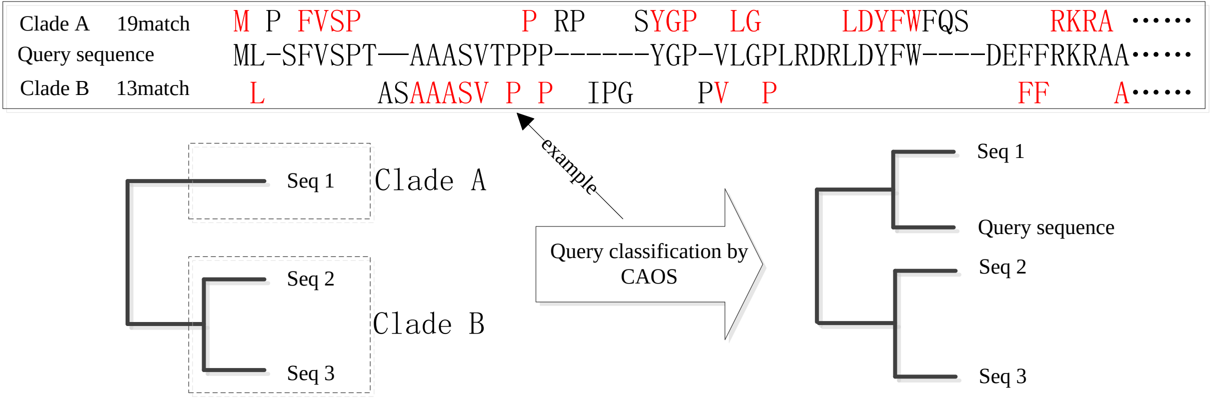

Clade A 19match
M P FVSP P RP SYGP LG LDYFWFQS RKRA ······
Query sequence
ML-SFVSPT—AAASVTPPP------YGP-VLGPLRDRLDYFW----DEFFRKRAA······
Clade B 13match
L ASAAASV P P IPG PV P FF A······
example
Seq 1
Clade A
Seq 2
Clade B
Seq 3
Query classification by CAOS
Seq 1
Query sequence
Seq 2
Seq 3
