# Supplementary material for: PlantOrDB: a genome-wide ortholog database for land plants and green algae
Source: BMC Plant Biol. 2015 Jun 26;15:161. doi: 10.1186/s12870-015-0531-4 (PMC4481079; doi:10.1186/s12870-015-0531-4)
Supplement: Additional file 6: Figure S6. — The web interface of Individual Gene Sequence-Annotation Viewer. [file 12870_2015_531_MOESM6_ESM.pdf]

Search Gene

Search by gene name | Search by PAC ID | Search | (e.g. GSVIVT01011697001, Lus10023737)

Gene Summary

PAC ID	17823493
Gene Name	GSVIVT01011697001
Species	Vitis vinifera
Homolog Family ID	1999
RBH Genes	Click Here

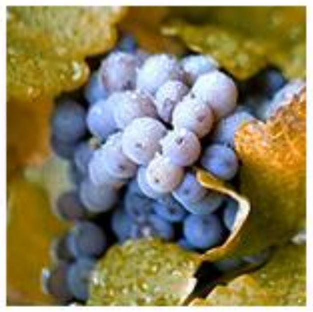

Gene Annotation

PAC ID	17823493
Locus Name	GSVIVG01011697001
Transcript Name	GSVIVT01011697001
Protein Name	GSVIVT01011697001
PFAM	PF04045
PANTHER	PTHR12058
KOG	KOG2826
KEGG EC	
KEGG Ortholog	K05758
GO	GO:0030833,GO:0005856
Best TAIR10 hit name	AT1G30825.1
Best TAIR10 hit symbol	ARPC2A,DIS2
Best TAIR10 hit defline	Arp2/3 complex, 34 kD subunit p34-Arc
Best rice hit name	
Best rice hit symbol	
Best rice hit defline	

Gene Sequence

Motif Search: Submit Reset

Next Previous

Rulers ☑

```
           10         20         30         40         50         60         70         80         90        100
0 MILLQSPSRF LLQTLLNRVQ NLEKGVELDY QWVEFDDVRY HVQVSMKYPQ FLLLSVSLPT PAQEAVFSGG LPFGAIEAIK ASYGVLVQIL DPPKDGFNLT
1 LKLNLSKLPP DEEYKHALLV KIASVREVVL GAPLRVVLKH LASRTVAPDI NRPFALVHRP KESFFLVPQA EKVTVVFPMR FKDSIDTVLA TSFLQEFVEA
2 RRTAGLNNAP PCLWSPSPPL ELRGTAGEAL SANAGFVTFV IFPRHVEGKK LDRTVWSLST FRAYVSYHVK CSEGFMHTRM RRRVESLIQA LDRAKPDLEK
3 SKKTVQGRSF KRLSLKEAHT NSNSQRWS*
```
